# Data of automated optical inspection of surface-mounted technology electronic production

**DOI:** 10.1016/j.dib.2024.110110

**Published:** 2024-01-30

**Authors:** Korbinian Pfab, Roman Eichler, Adarsh Mallandur, Marcel Rothering

**Affiliations:** a Department of Computer Science, Helsinki University, Finland; b Siemens AG, Erlangen, Germany

**Keywords:** Distribution drift, Quality inspection, Production environment, Soldering, Printed circuit board production, Machine learning, False call reduction, Real-world data

## Abstract

A popular soldering technique for printed circuit boards (PCB) is the so-called surface-mounted technology. After the soldering process an automated optical inspection (AOI) is the common method determining whether a PCB shall go to a manual inspection and rework station (MIS) or can directly go further to the next process step. Thereby, the AOI is a vision-based system deriving user defined physical measurements from a camera image. Based on these pre-defined measurements associated with static specification limits, the AOI labels each inspected soldering spot on a PCB as non-defect or defect. However, a large majority of PCBs are wrongly labelled defect, so-called false calls, causing a major manual labour effort at the MIS.

This dataset contains a 132-days recording of PCBs going through the MIS labelled as true defect or false call with the physical measurement by the AOI. Furthermore, the dataset may contain various distribution drifts of unknown type that can be explained by the high sensitivity of electronic production to small external factors that may change unrecognized and additionally the dataset has an unknown percentage of label error due the human labelling process.

Specifications Table

**Table:** 

Subject	Applied Machine Learning / Manufacturing Engineering
Specific subject area	False-call reduction of automated optical inspection in electronic production
Data format	Raw (anonymized)
Type of data	Tabular
Data collection	This dataset contains all points of a 132-day long recording phase of a real-world production of one production line of Siemens AG Germany. Thereby, only samples are included, that are sent from the AOI machine to the manual inspections. PCBs that are classified as good by the AOI machine are not considered. This results in a dataset with 440274 data points. Due to privacy concerns, all numerical features have been scaled between 0 and 1 and categorical values such as the error label was encoded in a categorical number.
Data source location	Siemens AG Bavaria Germany
Data accessibility	Repository name: Publication data: Data of automated optical inspection of surface-mounted technology electronic production Data identification number: 10.17632/99jzmh9658.1 Direct URL to data: https://data.mendeley.com/datasets/99jzmh9658/1

## 1 Value of the Data

• This dataset offers a recording from a real-world production of PCBs and their AOI. It can enable research on reducing the false-call ratio of AOI machines for instance with machine learning and enable researchers to compare their approaches.
• Researcher can test their machine learning and data science techniques on a real-world dataset that is highly imbalanced, drifts over time, has labelling error and is not generated by artificial experimental setting. Furthermore, it may be useful for any researcher that focuses on industrial applications of artificial intelligence and the challenges associated to its data.
• The dataset can be used for either developing advanced machine learning models for false call reduction [1], [2], [3], [4] or investigate the requirements of industrial application of artificial intelligence.

## 2 Data Description

**Fig. 1:**
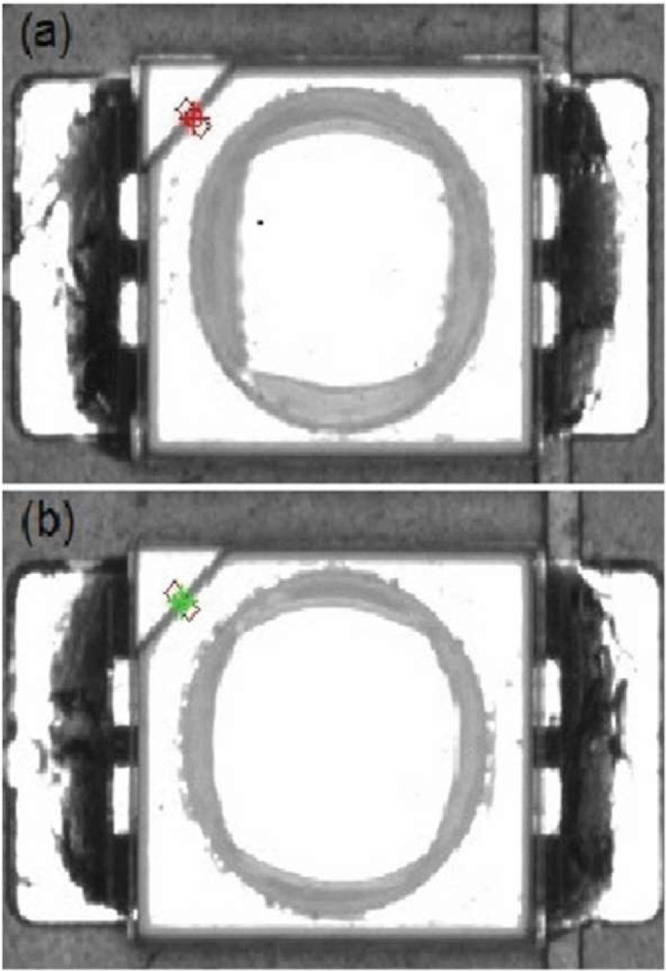
False call (a) and correct classified polarity (b) of a LED on different subpanels [1].

**Fig. 2:**
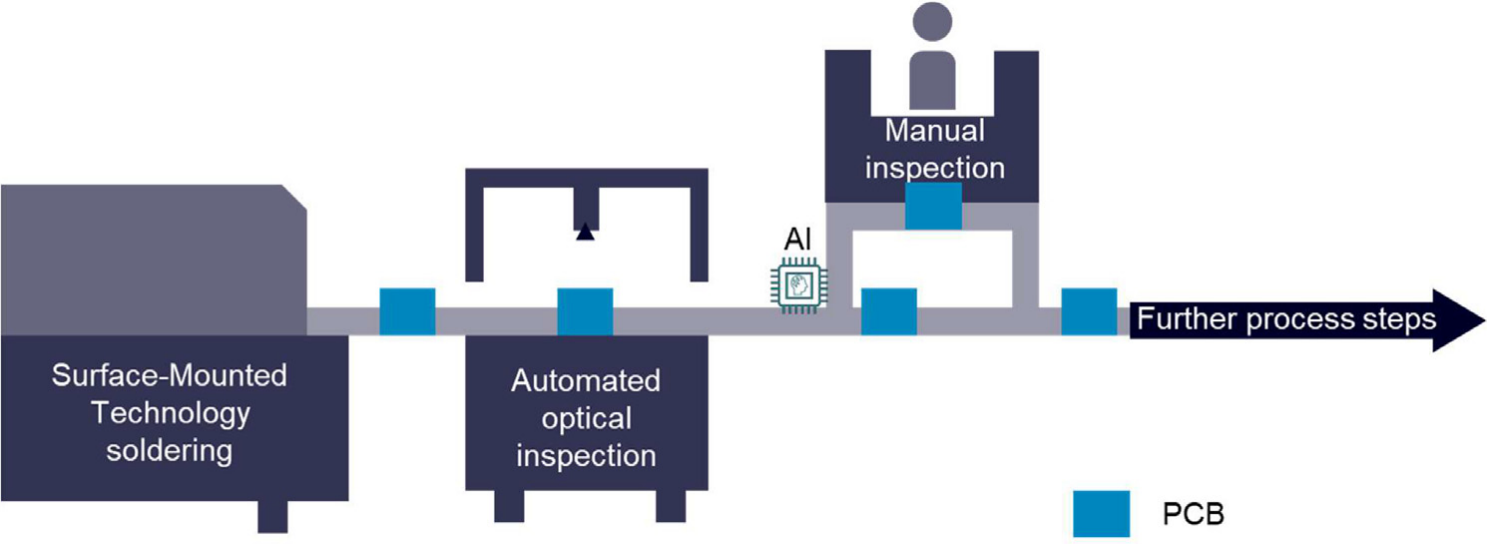
SMT electronic production scenario with additional AI-based decision gate.

As more and more devices are getting smart or even smarter, more printed circuit boards (PCB) are required to enable this smartness. Commonly, those PCBs are produced with the surface-mounted technology for soldering allowing to handle tiny components and minimal soldering spots. A whole production line also includes several other steps, like a quality inspection such as the automated optical inspection (AOI). Such machines evaluate the soldering outcome based on an image by deriving physical measurements and label the quality of the PCB either as non-defect or defect. While good PCBs go down straight to the subsequent production steps, bad PCBs are passed to a manual inspection and rework station (MIS). Even though there are specialized vendors of AOI machines, these machines tend to have a high rate of so-called false calls, i.e., non-defect boards that are labelled as defect and thus unnecessarily sent to the MIS, leading to high demand in manual work (cf. Fig. 1). Fig. 2: SMT electronic production scenario with additional AI-based decision gate Fig. 2 shows a visualization of this production process flows.

There exist already various publications [1], [2], [3], [4] about developing AI models for the reduction of false calls caused by the AOI machine. Yet, they lack of a common and public dataset since datasets of industrial applications are rarely published due to the fear of leaking expertise and secrets to the public. Thus, the results of those publications are not reproducible and comparable to each other. To enable this research for machine learning models to reduce the rate of false calls, we have recorded 132 days the features extracted by the AOI machine of PCBs sent to the manual inspection. By this publication, research on this use case can have a common baseline.

Our dataset is tabular. Each row represents the measurement values the AOI has measured for one soldering spot defined be the respective inspection type. Furthermore, the dataset consists in general of the following columns:

• **Timestamp**
  When an PCB enters the AOI, and the evaluation is starting a timestamp is generated. As multiple soldering spots and components are inspected for each PCB, multiple data points may have the same datapoint. It indicates a chronologicity and a grouping of several measurements.
• **Class**
  This column holds the label of each datapoint. Thereby, the label 0 is indicating a false call, i.e., a non-defect PCB labelled as defect by the AOI, and 1 is indicating a true defect, respectively. The class balance is highly imbalanced as can be seen in Table 1.

**Table 1:** Class distribution per inspection type.

Inspection type	Samples class 0	Samples class 1	Class Ratio
0	96735	318	0.003
1	56095	1578	0.028
2	126828	1346	0.011
3	150650	1281	0.009
4	5344	99	0.019
• **Inspection type**
  AOI machines in general can execute different measurements. Therefore, it is necessary that a technology expert configures where in on the PCB are soldering spots and components that need to be inspected in what ways for all different types of PCBs. These configurations are so-called inspection types and therefore encoded in this column with categorical values. The inspection type defines what measurements are contained by the *inspection_feat* columns. In this recording, five different configurations have been used.
• ***Meta*_feat columns**
  There are four columns starting with the tag *meta_feat* and an index*.* The information of those columns is persistent for all data points and contain information like the error code generated by the AOI machine, what component type is inspected, or with what angle the component is mounted on the PCB. All those columns are categorical.
• ***Inspection_feat* columns**
  There are 70 columns starting with the tag *inspection_feat* followed by an index. The contained information is generated by the AOI machine for its inspection based on an image and strongly depends on the inspection type since the measured physical unit may be different for every inspection type. For instance, for one inspection time the height of the soldering spot is measured and for another inspection type the misplacement is measured, but both measurement values are in the same column. Exemplary measurements contained in those columns are offset in x-coordinate, offset in y-coordinate, size of the soldering pad, if multiple soldering pads falsely connected, angle offset, or polarity of the component. Consequently, some columns can also be categorical or numerical within one inspection type. Furthermore, some columns could be empty for some inspection types. For those cases, the value zero is set by default. Nonetheless, the value zero also can be a valid measurement value in other cases. The mapping of available *inspection_feat* columns per inspection type can be seen in Fig. 3.

**Fig. 3:**
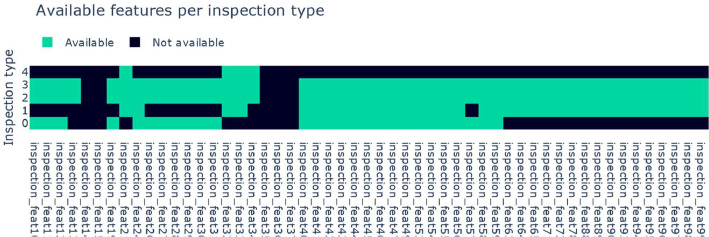
Visualization of available inspection features that are not available for each inspection type. 17 inspection features available for all features are not shown. If a feature is not available for an inspection type the value zero is set as default value.

As consequence, our dataset has 77 columns and contains 440274 samples. Furthermore, we want to mention that the dataset is drifting over time. The type of drift is unknown as well as how often drifts happened and when they appear.

An example notebook showing how to load and visualize the data can be found in [5]. The dataset table is available in [6] in the file *dataset.csv* and the inspection feature mapping can be found in the file *mapping.json*.

## 3 Experimental Design, Materials and Methods

**Fig. 4:**
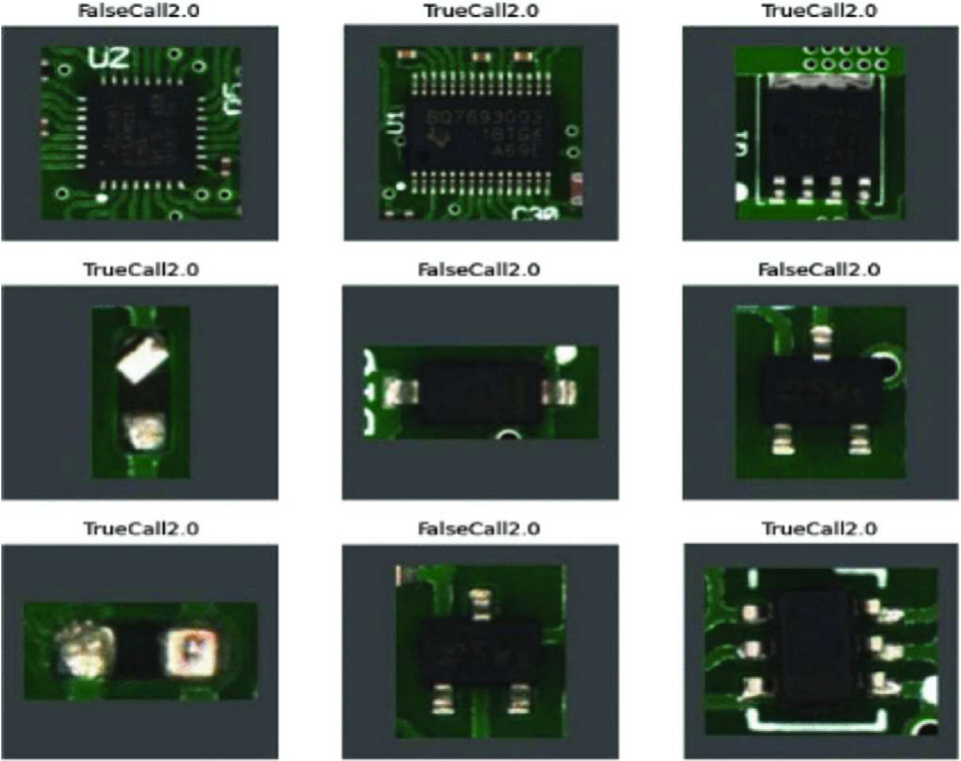
Raw images extracted by AOI [7].

This dataset is a recording of all PCBs classified as faulty by the AOI machine within the 132 days measurement phase of a real-world electronic production as shown in Fig. 2. Thereby, an AOI machine evaluates the quality of the PCB. For this, based on a given configuration, the inspection type, the AOI records raw images of specific components or soldering spots (cf. Figs. 1 and 4). From those images, then physical measurements like amongst others offset in x-coordinate, offset in y-coordinate, size of the soldering pad, if multiple soldering pads falsely connected, angle offset, or polarity of the component. What exactly measurements are extracted is defined by the inspection type as this is a reference to the used configuration. More insights on how AOI systems work can be found in [8,9].

**Fig. 5:**
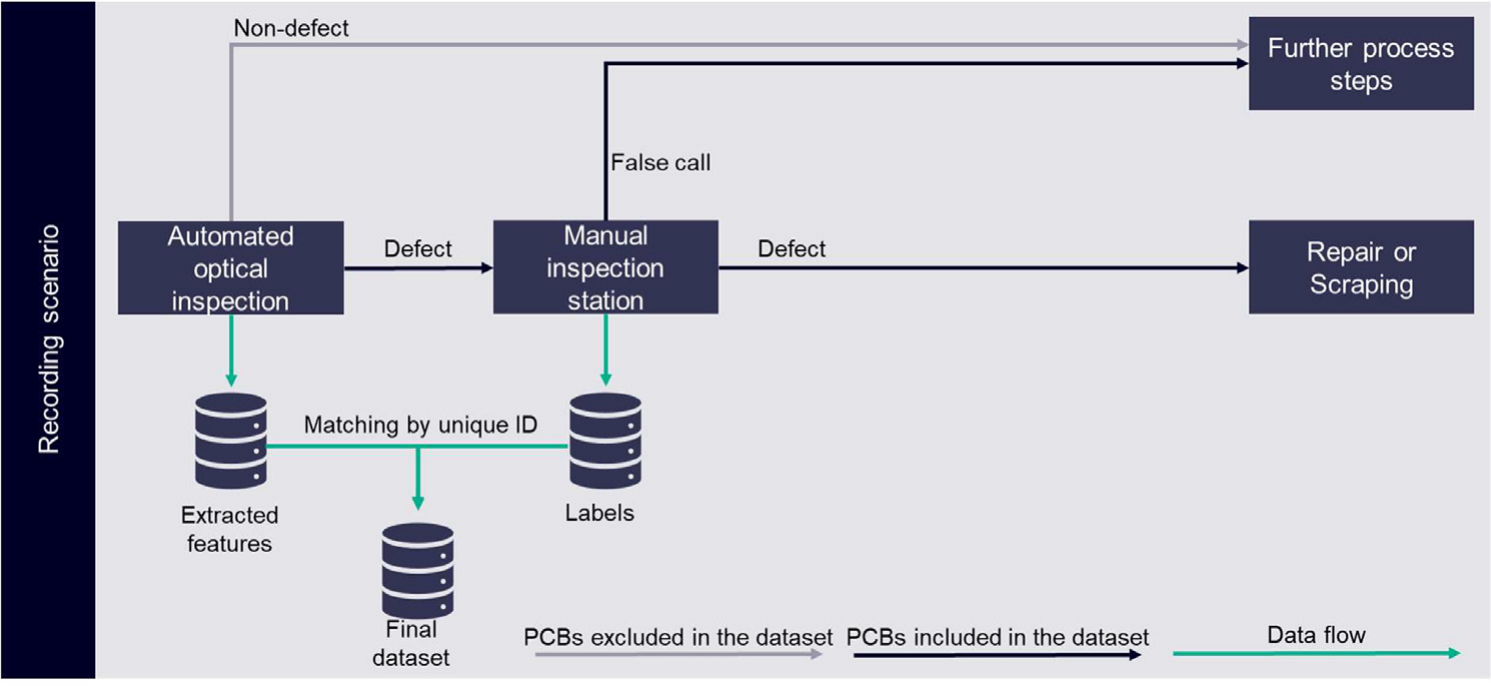
Recording scenario of this dataset.

Boards with defect soldering spots are forwarded to the manual inspection station while non-defect soldering spots are not considered in this dataset in any way. For gathering the data, we established a connection to the programable logic controller of the AOI machine containing the extracted features and of the manual inspection station to read the original log-files containing the label. The merge of both datasets was done by a unique identifier marking the PCB and the specific soldering spot. Besides our connection to the two stations, we did not apply any changes or experiment design to the shopfloor but left every condition as it is in the regular production flow. Thus, we have been able to record real-world conditions. Consequently, each label was created by one single operator at a time. Nevertheless, during the recording phase different operators were fulfilling this task. As consequence one must assume an error rate in labelling that may change over time. However, this error rate for labelling is unknown. The recording scenario can also be seen in Fig. 5.

Due to data privacy concerns, we must take the following measurement to anonymize the dataset before publishing it:

- Replacing the year of the timestamp with 1970.
- Scaling all *inspection_feat* columns between 0 and 1.
- Replacement of the original column names of all *inspection_feat* columns and *meta_feat* columns.
- Keeping the measured physical units in the *inspection_feat* columns unpublished.

## Limitations

We are aware of three possible limitations of the dataset. First, the label creation is based on human expertise and while the labels were done by multiple people, each label was done by just one person at a time. Thus, there is noise in the labels. Furthermore, the label classes are highly imbalanced. Finally, as this is a recording of real-world data, it might be that modification of the AOI machine program have been made that we are not aware of. This would cause distribution drifts, yet those could also have other reasons as the complete domain is quite sensitive and dynamic.

## Ethics Statement

Hereby, the author's state that we have read and followed the ethical requirements for publications in Data in Brief and confirm that the current work does not involve human subjects, animal experiments, or any data collected from social media.

## CRediT authorship contribution statement

**Korbinian Pfab:** Visualization, Writing – original draft, Writing – review & editing, Data curation. **Roman Eichler:** Methodology, Data curation, Validation. **Adarsh Mallandur:** Methodology, Data curation, Validation. **Marcel Rothering:** Writing – review & editing, Supervision.

## Data Availability

Publication data: Data of automated optical inspection of surface-mounted technology electronic production (Original data) (Mendeley Data).
